# Inhibition of p70 S6 kinase (S6K1) activity by A77 1726, the active metabolite of leflunomide, induces autophagy through TAK1-mediated AMPK and JNK activation

**DOI:** 10.18632/oncotarget.16737

**Published:** 2017-03-31

**Authors:** Xiulong Xu, §Jing Sun, Ruilong Song, Michelle E. Doscas, Ashley J. Williamson, Jingsong Zhou, Jun Sun, Xinan Jiao, Xiufan Liu, Yi Li

**Affiliations:** ^1^ Institute of Comparative Medicine, Yangzhou University, Yangzhou 225009, Jiangsu Province, P. R. China; ^2^ College of Veterinary Medicine, Yangzhou University, Yangzhou 225009, Jiangsu Province, P. R. China; ^3^ Department of Anatomy and Cell Biology Rush University Medical Center, Chicago, IL 60612, USA; ^4^ Core Facility, Yangzhou University, Yangzhou 225009, Jiangsu Province, P. R. China; ^5^ Rush Medical College, Chicago, IL 60612, USA; ^6^ Department of Physiology, Kansas City University of Medicine and Biosciences, Kansas City, MO 64106, USA; ^7^ Department of Medicine, University of Illinois at Chicago, Chicago, IL 60612, USA; ^8^ Jiangsu Key Laboratory of Zoonosis, Yangzhou University, Yangzhou 225009, Jiangsu Province, China; ^9^ Jiangsu Co-innovation Center for Prevention and Control of Important Animal Infectious Diseases and Zoonosis, Yangzhou University, Yangzhou 225009, Jiangsu Province, China; ^10^ Animal Infectious Disease Laboratory, College of Veterinary Medicine, Yangzhou University, Yangzhou 225009, China; ^11^ Lester and Sue Smith Breast Center, Baylor College of Medicine, Houston, TX 77030, USA

**Keywords:** p70 S6 kinase ULK, autophagy, leflunomide, mTOR

## Abstract

mTOR activation suppresses autophagy by phosphorylating ULK1 at S757 and suppressing its enzymatic activity. Here we report that feedback activation of mTOR in the PI-3 kinase pathway by two p70 S6 kinase (S6K1) inhibitors (PF-4708671 and A77 1726, the active metabolite of an immunosuppressive drug leflunomide) or by S6K1 knockdown did not suppress but rather induced autophagy. Suppression of S6K1 activity led to the phosphorylation and activation of AMPK, which then phosphorylated ULK1 at S555. While mTOR feedback activation led to increased phosphorylation of ULK1 at S757, this modification did not the disrupt ULK1-AMPK interaction nor dampen ULK1 S555 phosphorylation and the induction of autophagy. In addition, inhibition of S6K1 activity led to JNK activation, which also contributed to autophagy. 5Z-7-oxozeaenol, a specific inhibitor of TAK1, or TAK1 siRNA blocked A77 1726-induced activation of AMPK and JNK, and LC3 lipidation. Taken together, our study establishes S6K1 as a key player in the PI-3 kinase pathway to suppress autophagy through inhibiting AMPK and JNK in a TAK1-dependent manner.

## INTRODUCTION

Macroautophagy (referred as autophagy hereafter) is a highly conserved catabolic process characterized by the formation of the double-membraned vesicles (autophagosomes), fusion with lysosomes, and degradation of cellular materials. Autophagy is activated primarily by nutrient and energy stress. Other autophagy inducers include hypoxia, anticancer drugs, damaged organelles, protein aggregates, and infectious agents. AMP-activated protein kinase (AMPK) and mechanistic target of rapamycin (mTOR) sense energy stress and nutrient depletion, respectively, and play pivotal roles in regulating autophagy [1, 2]. AMPK directly phosphorylates ULK1 at S555 and activates it or indirectly activates ULK1 by inhibiting mTORC1 activity [3–5]. mTOR, a serine/threonine kinase that interacts with several adaptor proteins to form the mTOR complex 1 (mTORC1), phosphorylates ULK1/2 at serine 757 (S757), disrupts its interaction with AMPK and prevents it from activating the autophagy pathway [6, 7]. Inactivation of mTORC1 by nutrient insufficiency or by rapamycin, an inhibitor of mTOR, induces autophagy [6, 7]. S6K1 is a serine/threonine kinase downstream of mTORC1. S6K1 plays important roles in cancer, diabetes, obesity, and ageing [8]. S6K1 depletion mimics the effect of diet restriction [9, 10]. Interestingly, AMPK activity is up-regulated in the skeletal muscle tissues and myotubes of S6K1-deficient mice due to elevated AMP/ATP ratios [9, 11]. Sch9, an equivalent of mammalian S6K1 in yeast, has been reported to inhibit autophagy [12, 13]. Whether mammalian S6K1 also suppresses autophagy is incompletely understood.

TAK1 is a serine/threonine kinase that plays a crucial role in regulating cell survival, differentiation, apoptosis, and inflammatory responses. TAK1 is activated by IL-1 and TGF-β receptors, Toll-like receptors (TLR), CD40, and the B cell receptor [14–16]. TAK1 is involved in activating several intracellular kinases, including p38, JNK, and I-kappa B kinase complex (IKK) [17–20]. TAK1 plays a critical role in activating the tumor suppressor protein LKB1, and AMPK T172 phosphorylation is inhibited in TAK1-deficient embryos and in TAK1-deficient embryonic fibroblast cells [21]. Herrero-Martin et al. reported that TAK1 plays a critical role in tumor necrosis factor-related apoptosis-inducing ligand (TRAIL)-induced AMPK activation [22]. Inokuchi-Shimizu et al. [23] reported that TAK1 is required for starvation-induced AMPK and ULK1 phosphorylation and activation, and plays a critical role in inducing autophagy. Moreover, TAK1 deficiency partially blocks rapamycin-induced autophagy in hepatocytes [23]. These observations strongly suggest that autophagy induced by mTOR suppression is in part mediated through TAK1. The underlying molecular mechanisms remain to be defined.

A77 1726 is the active metabolite of leflunomide (Arava™), an anti-inflammatory drug primarily used for treating rheumatoid arthritis. Mechanistic studies revealed that A77 1726 and its parental drug, leflunomide, are capable of inhibiting tyrosine phosphorylation and pyrimidine nucleotide synthesis [24–31]. The ability of A77 1726 to inhibit the activity of dihydroorotate dehydrogenase (DHO-DHase), a rate-limiting enzyme in pyrimidine nucleotide synthesis, is much stronger than its ability to inhibit the activity of protein tyrosine kinases such as p56^lck^, p59^fyn^, and PDGF receptor [24–28]. Recently, we reported that A77 1726 and its parental drug, leflunomide, inhibit the activity of S6K1 in an *in vitro* kinase assay and in cell culture, and that inhibition of S6K1 activity by A77 1726 leads to the feedback activation of the PI-3 kinase pathway [32]. Here we report that mTOR feedback activation by A77 1726 or PF-4708671 did not inhibit but rather induced autophagy. We also found that A77 1726-induced autophagy was mediated through inhibiting S6K1 activity, subsequently leading to activation of AMPK and JNK through TAK1, and that activation of AMPK and JNK both contributed to A77 1726-induced autophagy.

## RESULTS

### A77 1726 induces autophagy

**Figure 1:**
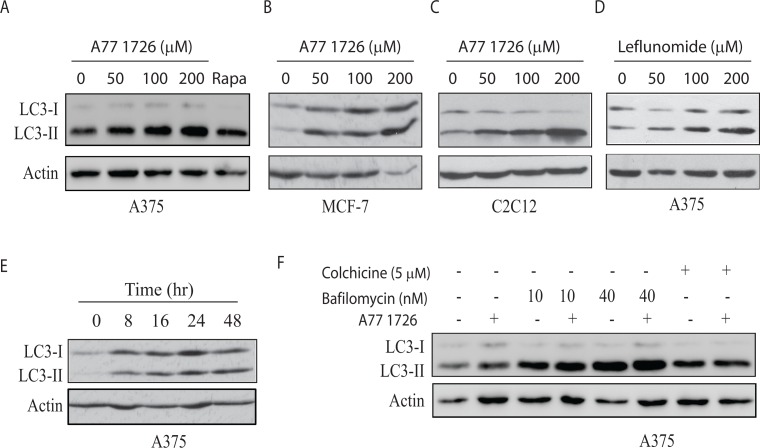
A77 1726 increases LC3-II expression **(A-C)** Dose-dependent increase of LC3-II levels. A375 **(A)**, MCF-7 **(B)**, and C2C12 **(C)** cells were incubated in complete DMEM medium in the absence or presence of the indicated concentrations of A77 1726 for 16 hr. Rapamycin (Rapa) (20 nM) was included as a control. LC3 and actin expression was analyzed by Western blot. **(D)** A375 cells were incubated in complete DMEM medium in the absence or presence of the indicated concentrations of leflunomide for 16 hr. LC3 and actin expression were analyzed by Western blot. **(E)** Time-dependent increase of LC3-II lipidation. A375 cells were incubated in the presence of A77 1726 (200 μM) for the indicated time. Cell lysates were analyzed for LC3 and actin levels by Western blot. **(F)** The effect of bafilomycin and colchicine. A375 cells seeded in 6-well plates were incubated in complete DMEM medium in the absence or presence of bafilomycin (10 or 40 nM) or colchicine (5 μM) for 16 hr. Cell lysates were analyzed for LC3 and actin expression by Western blot.

**Figure 2:**
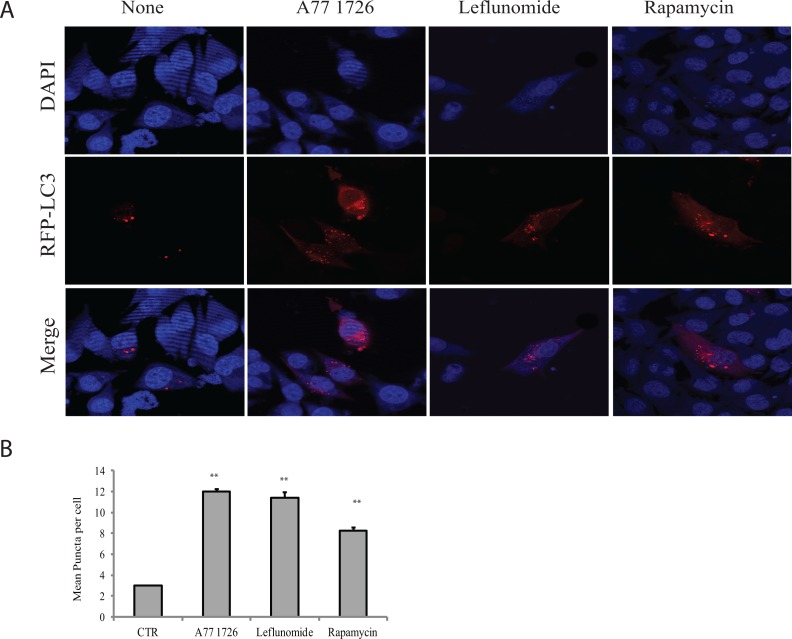
Induction of autophagosomes by A77 1726 A375 cells were transfected with the expression vector pmLC3-RFP. The cells were left untreated or treated with A77 1726 (200 μM), rapamycin (20 nM), or leflunomide (200 μM) for 16 hr. Autophagosomes were visualized under a confocal microscope **(A)**. The puncta of autophagosomes were counted under a fluorescence microscope and plotted in a bar graph with statistical analysis **(B)**. **p<0.01, compared to the control.

Our recent study showed that A77 1726 suppresses S6K1 activity and subsequently induces feedback activation of PI3K, AKT, and mTOR in A375 cells [32]. Since mTOR activation suppresses autophagy [6], we tested if mTOR feedback activation by A77 1726 also suppressed autophagy. Unexpectedly, A77 1726 induced LC3-II lipidation in a dose-dependent manner in A375 (Figure 1A), MCF-7 breast cancer cells (Figure 1B), and C2C12 myotubes (Figure 1C). Rapamycin included as a positive control was less effective than A77 1726 to increase LC3-II levels in A375 cells (Figure 1A). Leflunomide, the parental drug of A77 1726, increased LC3-II levels too in A375 cells in a dose-dependent manner (Figure 1D). Increased LC3-II lipidation could be observed 8 hr after the addition of A77 1726 and lasted up to 48 hr in A375 cells (Figure 1E). Confocal microscopic fluorescence analysis revealed that LC3 formed autophagosomes in A375 cells in the presence of A77 1726, leflunomide, or rapamycin (Figure 2A). Enumeration of autophagosomes showed that A77 1726, leflunomide, and rapamycin all significantly increased the number of puncta (Figure 2B). Increased numbers of autophagosome puncta were also observed in MCF-7 cells treated with A77 1726, leflunomide, or rapamycin (data not shown). To determine if increased LC3-II lipidation was due to the stall of autophagy flux or was indeed due to the induction of autophagy, we tested the effect of bafilomycin and colchicine on A77 1726-induced autophagy. As shown in Figure 1F, A77 1726, bafilomycin or colchicine alone increased the levels of both LC3-I and LC3-II. Combination of A77 1726 with bafilomycin or colchicine further increased the ratio of LC3-II to LC-I, compared to bafilomycin or colchicine alone. These results suggest that A77 1726 induces autophagy, and that increased LC3-II levels are not due to the inhibition of the autophagy flux.

**Figure 3:**
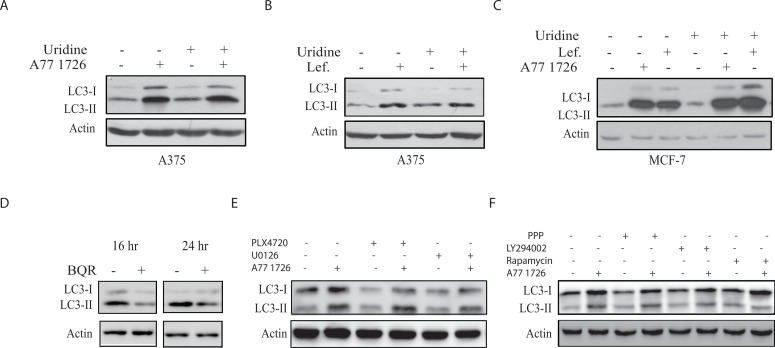
A77 1726 increases LC3-II levels independent of pyrimidine nucleotide depletion and the feedback activation of the PI-3 and MAP kinase pathways **(A&B)** A375 cells seeded in a 6-well plate were incubated in complete DMEM medium in the absence or presence of A77 1726 (200 μM) **(A)** or leflunomide (200 μM) **(B)** and/or uridine (200 μM) for 16 hr. Cells were harvested and analyzed for LC3 and actin expression by Western blot. **(C)** MCF7 cells were similarly treated as in A and B and analyzed for LC3 and actin levels. **(D)** The effect of brequinar sodium (BQR) on LC3 lipidation. A375 cells seeded in a 6-well plate were incubated in complete DMEM medium in the absence or presence of BQR (10 μM) for 16 or 24 hr. LC3 and actin levels were analyzed by Western blot. **(E)** A375 cells seeded in 6-well plates were pre-treated with vehicle (0.1% dimethyl sulfoxide), PLX4720 (1 μM) or U0126 (10 μM) for 1 hr, followed by addition of A77 1726 (200 μM) and incubation for 16 hr. Cells were harvested and analyzed for LC3 and actin expression. **(F)** A375 cells seeded in 6-well plates were pre-treated with vehicle (0.1% dimethyl sulfoxide), PPP (1 μM), LY294002 (10 μM) or rapamycin (20 nM) for 1 hr, followed by addition of A77 1726 (200 μM) and incubation for 16 hr. Cells were harvested and analyzed for LC3 and actin expression.

As an inhibitor of DHO-DHase, A77 1726 inhibits pyrimidine nucleotide synthesis [33]. To determine if increased LC3-II lipidation was due to pyrimidine nucleotide depletion, we tested whether exogenous uridine blocked A77 1726-induced LC3-II lipidation. According to our previous studies, exogenous uridine added into rapidly proliferating cells or injected into mice can be readily uptaken by cells and normalize intracellular pyrimidine nucleotide levels [24, 26] Uridine (200 μM) itself had no effect on LC3-II levels and did not block A77 1726- (Figure 3A) or leflunomide-induced (Figure 3B) LC3 lipidation in A375 cells. Uridine had also no effect on A77 1726- or leflunomide-induced LC3-II lipidation in MCF-7 cells (Figure 3C). Moreover, brequinar sodium (BQR), a potent inhibitor of pyrimidine nucleotide synthesis, did not increase but rather slightly decreased LC3-II lipidation (Figure 3D).

### A77 1726-induced autophagy is independent of the feedback activation of the PI-3 and MAP kinase pathways

Our recent study showed that A77 1726 induces the feedback activation of the PI-3 and MAP kinase pathways; and that PLX4720, an inhibitor of Raf kinase, and U0126, a MEK inhibitor, block A77 1726-induced phosphorylation of ERK1/2^T202/Y204^ and MEK1/2^S217/S221^ [32]. Here we found that these inhibitors did not change the levels of LC3-II lipidation in the presence of A77 1726 (Figure 3E). The IGF-1 receptor tyrosine kinase is responsible for S6K1-mediated negative feedback activation of the MAP and PI-3 kinase pathways [34]. As shown in Figure 3F, PPP, an inhibitor of IGF-1 receptor; LY294002, a PI-3 kinase inhibitor, and rapamycin, an inhibitor of mTOR, were unable to block A77 1726-induced LC3-II increase (Figure 3F).

### Suppression of S6K1 activity induces autophagy

**Figure 4:**
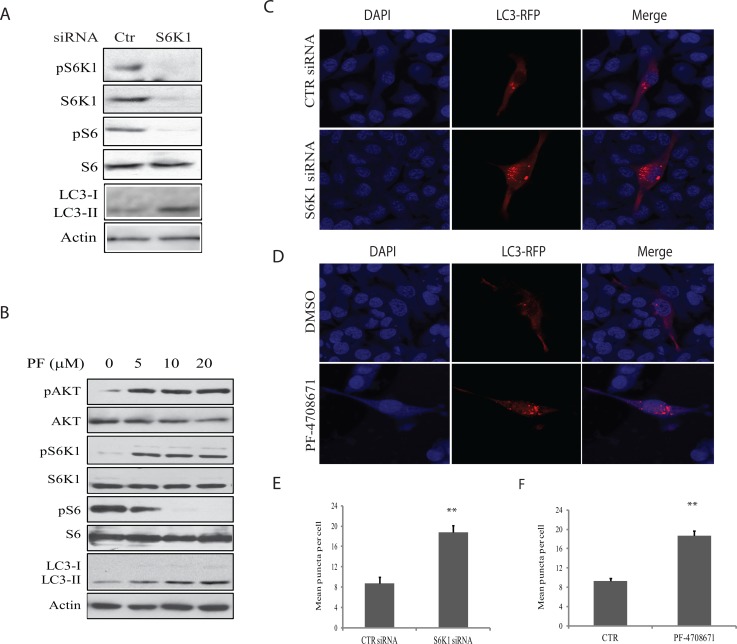
Role of S6K1 in autophagy **(A)** The effect of S6K1 knockdown on LC3-II lipidation.A375 cells were transfected with scrambled or S6K1 siRNA (2.5 nmole each). After incubation for 48 hr, the cells were harvested and analyzed for S6K1 expression and phosphorylation of the indicated proteins by Western blot. **(B)** The effect of the S6K1 inhibitor on LC3-II expression.A375 cells seeded in a 6-well plate were incubated in complete DMEM medium in the absence or presence of the indicated concentrations of PF-4708671 for 16 hr. Cells were harvested and analyzed for LC3 and actin expression by Western blot. **(C)** The effect of S6K1 knockdown on autophagosome formation. A375 cells seeded on the coverslips were first transfected with scrambled or S6K1 siRNA (2.5 nmole each). After incubation overnight, the cells were transfected with pmLC3-RFP expression vector. After incubation for 48 hr, the cells were fixed in methanol and visualized for autophagosomes under a fluorescent microscope. **(D)** The effect of the S6K1 inhibitor on LC3-II expression.A375 cells seeded on coverslips were transfected with LC3-RFP expression vector. After incubation for 24 hr, the cells were treated with PF-4708671 (10 μM) for 16 hr. Cells were fixed in methanol and visualized for autophagosomes under a fluorescence microscope. **(E & F)** The puncta of autophagosomes were counted under a fluorescent microscope plotted in a bar graph with statistical analysis. **p<0.01, compared to the control.

To investigate the role of S6K1 in mediating A77 1726-induced autophagy, we first examined the effect of S6K1 knockdown on autophagy in A375 cells. As shown in Figure 4A, S6K1 siRNA very effectively silenced S6K1 expression. S6K1 knockdown led to decreased S6 phosphorylation but increased the levels of LC3-II. Consistently, S6K1 knockdown also led to the increase of the number of LC3-RFP puncta (Figure 4C & 4E). Similar to A77 1726, PF-4708671, a specific inhibitor of S6K1, inhibited S6 phosphorylation but induced the feedback activation of the PI-3 kinase pathway, as shown by increased AKT and S6K1 phosphorylation (Figure 4B). Consistently, PF-4708671 induced LC3 lipidation in a dose-dependent manner in A375 cells (Figure 4B) and increased the number of LC3-RFP puncta (Figure 4D & 4F).

### A77 1726 induces AMPK and ULK1 phosphorylation by inhibiting S6K1 activity

**Figure 5:**
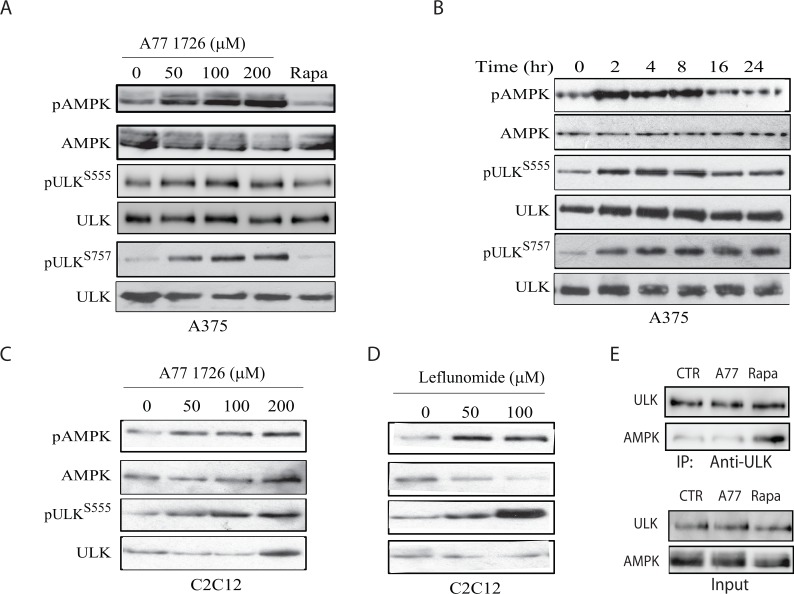
A77 1726 induces AMPK and ULK1 phosphorylation **(A & B)** Time- and dose-dependent induction of AMPK and ULK1 phosphorylation by A77 1726. A375 cells were incubated in complete DMEM medium in the absence or presence of the indicated concentrations of A77 1726 for 16 hr **(A)** or in the presence of A77 1726 (200 μM) for the indicated time **(B)**. **(C & D)** Dose-dependent induction of AMPK and ULK1 phosphorylation by A77 1726 **(C)** or leflunomide **(D)** in C2C12 myotubes. C2C12 cells were seeded in 6-well plates and differentiated into myotubes. Myotubes were treated with the indicated concentrations of A77 1726 or leflunomide for 16 hr. Cell lysates were prepared and analyzed by Western blot with the indicated antibodies. **(E)** A77 1726 does not disrupt AMPK and ULK1 interaction. A375 cells were incubated in the absence or presence of A77 1726 (200 μM) or rapamycin (20 nM) for 16 hr. Cell lysates were immunoprecipitated with an anti-ULK1 antibody, followed by Western blot analysis with an anti-ULK1 or anti-AMPK antibody. Unimmunoprecipitated cell lysates were included as input controls.

ULK1 is phosphorylated at S555 and activated by AMPK [3, 35]. Previous studies have shown that AMPK is activated in S6K1-deficient mice [9, 11]. A77 1726 may induce autophagy by inhibiting S6K1 activity and subsequent activation of AMPK and then ULK1. Indeed, we found that A77 1726 induced AMPK phosphorylation at T172 and ULK1 phosphorylation at S555 in A375 cells in a dose- (Figure 5A) and time-dependent (Figure 5B) manner. mTOR phosphorylates ULK1 at S757 and inhibits its activity as well as autophagy [6, 7]. mTOR is activated by A77 1726 due to the feedback activation of the PI-3 kinase pathway [32]. As expected, A77 1726 induced ULK1 phosphorylation at S757 in A375 cells in a dose- (Figure 5A) and time-dependent (Figure 5B) manner. Rapamycin had little effect on AMPK^T172^ and ULK1^S555^ phosphorylation but inhibited ULK1^S757^ phosphorylation (Figure 5A). Consistently, A77 1726 and leflunomide induced AMPK^T172^ and ULK1^S555^ phosphorylation in C2C12 cells in a dose-dependent manner (Figure 5C & 5D). ULK1 S757 phosphorylation disrupts AMPK and ULK1 interaction [6]. We tested whether mTOR feedback activation and ULK1 S757 phosphorylation by A77 1726 inhibited the formation of the AMPK-ULK complex. As shown in Figure 5E, the levels of ULK1 in anti-AMPK immunoprecipitate of A77 1726-treated A375 cells were equal to those in the untreated control. In contrast, inhibition of ULK1 S757 phosphorylation by rapamycin led to increased AMPK and ULK1 interaction (Figure 5E).

### The effect of the feedback activation of the PI-3 and MAP kinase pathways on A77 1726-induced AMPK and ULK1 phosphorylation

**Figure 6:**
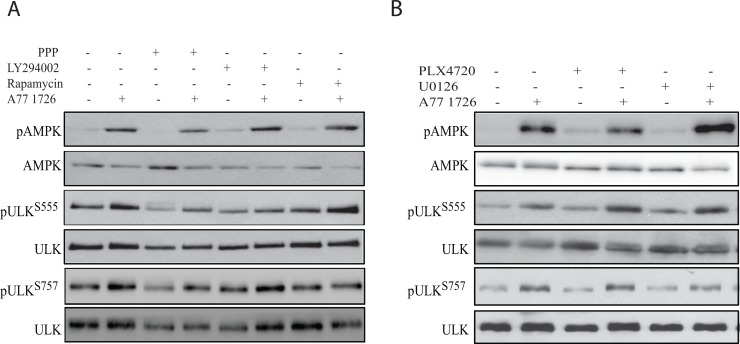
A77 1726-induced AMPK and ULK1 phosphorylation is independent of the feedback activation of the PI-3 and MAP kinase pathways **(A)** A375 cells seeded in 6-well plates were pre-treated with vehicle (0.1% dimethyl sulfoxide), PPP (1 μM), LY294002 (10 μM), or rapamycin (20 nM) for 1 hr, followed by addition of A77 1726 (200 μM) and incubation for 16 hr. Cells were harvested and analyzed for AMPK and ULK1 phosphorylation. **(B)** A375 cells seeded in 6-well plates were pre-treated with vehicle (0.1% dimethyl sulfoxide), PLX4720 (1 μM), U0126 (10 μM), or PD98059 (10 μM) for 1 hr, followed by addition of A77 1726 (200 μM) and incubation for 16 hr. Cells were harvested and analyzed for ULK and AMPK phosphorylation with indicated antibodies.

We first determined the effect of the feedback activation of the PI-3 kinase pathway on AMPK and ULK1 phosphorylation. As shown in Figure 6A, all three inhibitors of the PI-3 kinase pathway, including the IGF-1 receptor (PPP), PI-3 kinase (LY294002), and mTOR (rapamycin), did not significantly block A77 1726-induced AMPK phosphorylation. All three inhibitors had little effect on A77 1726 induced ULK1 phosphorylation at S555. In contrast, all three inhibitors almost completely inhibited A77 1726-induced ULK1 phosphorylation at S757. These observations confirmed that increased ULK1 phosphorylation at S757 is indeed mediated by feedback activation of the PI-3 kinase pathway through mTOR. We next determined if feedback activation of the MAP kinase pathway was involved in A77 1726-induced AMPK and ULK1 phosphorylation. As shown in Figure 6B, PLX4720, an inhibitor of B-Raf kinase, and U0126, an inhibitor of MEK kinase, had little or no effect in A77 1726-induced AMPK and ULK1 phosphorylation at both S555 and S757.

### Role of S6K1 in regulating AMPK and ULK1 phosphorylation

**Figure 7:**
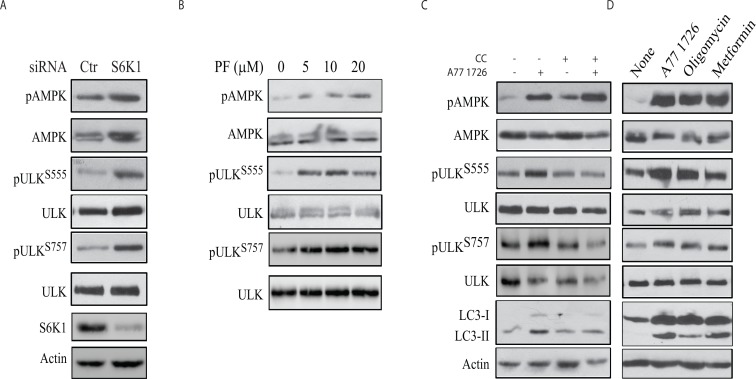
The role of AMPK in A77 1726-induced autophagy **(A & B)** S6K1 knockdown and PF-4708671 induces AMPK and ULK1 phosphorylation. **(A)** A375 cells were transfected with S6K1 siRNA. After incubation for 48 hr, the cells were harvested and analyzed for AMPK and ULK1 phosphorylation with indicated antibodies. **(B)** A375 cells were treated with the indicated concentrations of PF-4708671 for 16 hr and analyzed for AMPK and ULK1 phosphorylation. **(C)** Inhibition of AMPK and ULK1 phosphorylation and LC3 expression by compound C (CC). A375 cells were incubated in the absence or presence of A77 1726 (200 μM) and/or CC (1 μM) for 16 hr. Cell lysates were prepared and analyzed for ULK and AMPK phosphorylation, and for LC3 and actin expression by Western blot. **(D)** The effect of AMPK activators on AMPK and ULK1 phosphorylation and LC3 expression. A375 cells were incubated in the absence or presence of A77 1726 (200 μM), oligomycin (10 μM) or metformin (5 mM) for 16 hr. Cell lysates were prepared and analyzed by Western blot with the indicated antibodies.

To confirm that A77 1726-induced AMPK and ULK1 phosphorylation was mediated by inhibiting S6K1 activity, we tested if S6K1 knockdown or inhibition of S6K1 activity by PF-4708671 also led to increased AMPK and ULK1 phosphorylation. As shown in Figure 7A, suppression of S6K1 expression led to increased AMPK and ULK1 phosphorylation at S555 and S757. PF-470867 induced AMPK phosphorylation in a dose-dependent manner (Figure 7B). Due to its strong ability to induce the feedback activation of the PI-3 kinase pathway, PF-470867 induced ULK1 phosphorylation at S555 and S757 even at low concentrations (Figure 7B). These results collectively suggest that inhibition of S6K1 activity plays a critical role in inducing AMPK and ULK1 phosphorylation.

### AMPK mediates A77 1726-induced ULK1 phosphorylation and autophagy

To confirm that the effect of A77 1726 on ULK1 phosphorylation was indeed mediated through AMPK, we first tested if compound C (CC), an inhibitor of AMPK, was able to block A77 1726-induced ULK1 phosphorylation and LC3 expression. As shown in Figure 7C, CC had no effect on A77 1726-induced AMPK phosphorylation but blocked A77 1726-induced ULK1 phosphorylation at ULK1 S555 and LC3-II lipidation. CC blocked A77 1726-induced ULK1 S757 phosphorylation, probably as the result of inhibition of raptor phosphorylation and inhibition of mTOR activity [36]. In contrast, two AMPK activators, oligomycin and metformin, induced AMPK and ULK1 S555 phosphorylation (Figure 7D). Both oligomycin and metformin induced LC3 lipidation and slightly increased ULK1 S757 phosphorylation. These observations collectively suggest that A77 1726 induces autophagy by AMPK activation-induced ULK1 phosphorylation.

### A77 1726 induces p62 expression

**Figure 8:**
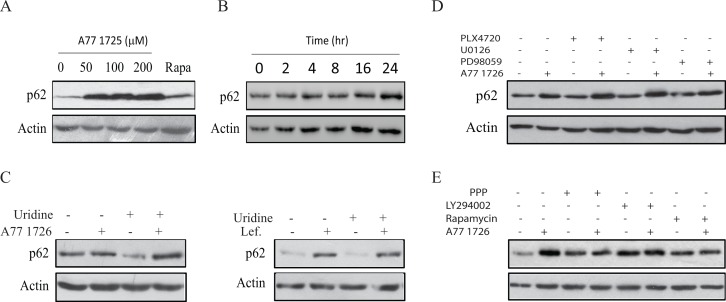
A77 1726 induces p62 expression **(A & B)** Dose- and time-dependent induction of p62 by A77 1726.A375 cells were treated with the indicated concentration of A77 1726 or rapamycin (20 nM) **(A)** for 16 hr or treated with A77 1726 (200 μM) for the indicated time **(B)**. Cell lysates were prepared and analyzed for p62 and actin expression. **(C-E)** A375 cells were treated with A77 1726 (200 μM) or leflunomide in the absence or presence of uridine **(C)**, MAP kinase pathway inhibitors **(D)**, or the PI-3 kinase inhibitors **(E)** for 16 hr. Cells were harvested and analyzed for p62 and actin levels by Western blot.

p62 is a ubiquitin-binding protein that sequesters ubiquitinated proteins for lysosomal degradation through interacting with LC3-II in autophagosomes [37]. The p62 level is usually decreased following autophagy induction. However, p62 expression is induced by resveratrol, an autophagy inducer, by transcriptional up-regulation through the MAP kinase-activated AP1 transcription factor [38]. As shown in Figure 8A, A77 1726 increased p62 levels after a prolonged exposure for 24 hr. Rapamycin slightly induced p62 expression. A77 1726-induced p62 expression was time-dependent (Figure 8B). Induction of p62 by A77 1726 or leflunomide was not due to its effect on pyrimidine nucleotide synthesis, since exogenous uridine was unable to block A77 1726- or leflunomide-induced p62 expression (Figure 8C). The inhibitors of the MAP kinase pathway, including PLX4720, U0126, and PD98059, had no effect on A77 1726-induced p62 expression (Figure 8D). The inhibitors of the PI-3 kinase pathway, including PPP, LY294002, and rapamycin, did not increase A77 1726-induced p62 expression (Figure 8E).

### JNK activation is required for A77 1726-induced p62 expression

**Figure 9:**
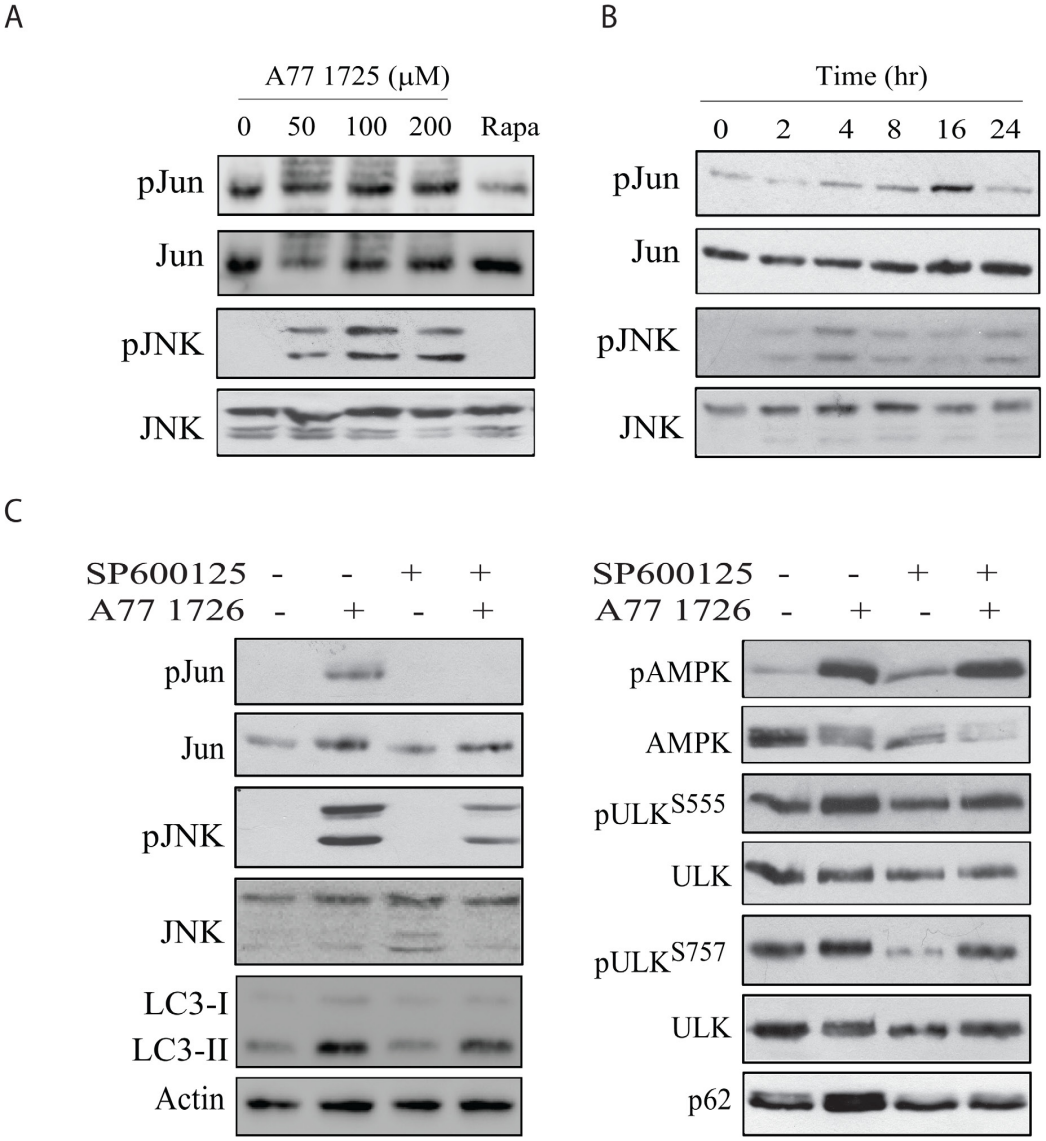
JNK activation is required for A77 1726-induced p62 expression A375 cells were treated with the indicated concentration of A77 1726 or rapamycin (20 nM) **(A)** for 16 hr or treated with A77 1726 (200 μM) for the indicated time **(B)**. **(C)** A375 cells were treated in the absence or presence of A77 1726 (200 μM) and/or SP6000125 (10 μM) for 16 hr. Cell lysates were prepared and analyzed for the protein levels with the indicated antibodies.

An earlier study showed that activation of JNK is required for the increased p62 expression by resveratrol in colorectal cancer cell lines [38, 39]. Here we tested whether A77 1726 was able to activate JNK, subsequently leading to increased p62 expression. A77 1726 induced JNK and Jun phosphorylation in a time- and dose-dependent manner (Figure 9A & 9B). SP600125, a specific inhibitor of JNK, blocked A77 1726-induced Jun and JNK phosphorylation. SP600125 also blocked p62 expression and LC3-II lipidation but had no effect on A77 1726-induced AMPK and ULK1 phosphorylation (Figure 9C). These results suggest that JNK activation is responsible for A77 1726-induced p62 expression.

**Figure 10:**
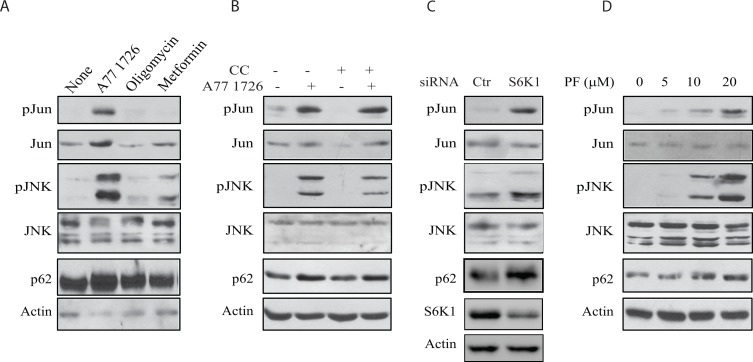
JNK activation is required for A77 1726-induced p62 expression **(A)** The effect of AMPK activators on JNK activation. A375 cells were incubated in the absence or presence of A77 1726 (200 μM), oligomycin (10 μM), or metformin (5 mM) for 16 hr. Cells were harvested and analyzed with the antibodies against phospho-JNK, phospho-Jun, p62 and actin. **(B)** A375 cells were incubated in the absence or presence of A77 1726 (200 μM) and/or compound C (CC) (1 μM) for 16 hr. Cell lysates were prepared and analyzed for ULK and AMPK phosphorylation, and for LC3 and actin expression by Western blot. **(C)** A375 cells were transfected with S6K1 siRNA. After incubation for 48 hr, the cells were harvested and analyzed for Jun, JNK, p62, and actin expression. **(D)** A375 cells were treated with the indicated concentrations of PF-4708671 for 16 hr. Cells were harvested and analyzed for Jun, JNK, p62, and actin expression.

The inability of SP600125 to block A77 1726-induced AMPK and ULK1 phosphorylation suggests that AMPK is not involved in A77 1726-induced JNK activation. As shown in Figure 10A, oligomycin and metformin, two AMPK activators, had no or little effect on JNK and Jun phosphorylation nor induced p62 expression. Compound C, an AMPK inhibitor, was unable to block A77 1726-induced JNK and Jun phosphorylation as well as p62 expression (Figure 10B). S6K1 knockdown led to increased JNK and Jun phosphorylation as well as increased p62 expression (Figure 10C). Consistently, PF-4708671 induced JNK and Jun phosphorylation as well as p62 expression in a dose-dependent manner (Figure 10D). These observations collectively suggest that JNK activation by A77 1726 is mediated by inhibition of S6K1 activity.

### Role of TAK1 in S6K1-mediated regulation of autophagy

**Figure 11:**
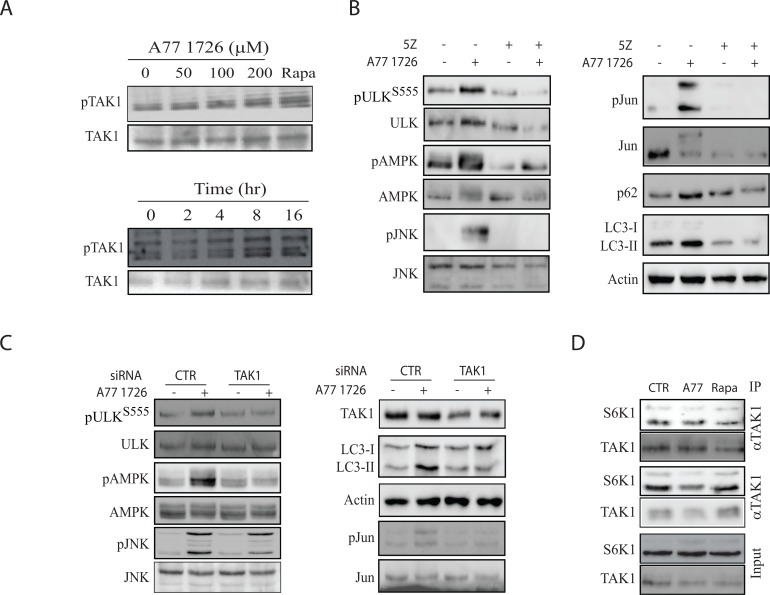
TAK1 mediates A77 1726-induced AMPK and JNK activation **(A)** A77 1726 induces TAK1 phosphorylation. A375 cells were incubated in the absence or presence of the indicated concentrations of A77 1726 or rapamycin (20 nM) or A77 1726 (200 μM) for the indicated time. Cell lysates were analyzed with an anti-TAK1^T184/187^ phosphorylation antibody, followed by re-probing with an anti-TAK1 antibody. **(B)** The effect of the TAK1 inhibitor on protein phosphorylation and LC3-II levels.A375 cells were treated with A77 1726 (200 μM) and/or 5Z (5 μM) for 16 hr. Cell lysates were analyzed for the phosphorylation of indicated proteins by Western blot. **(C)** The effect of TAK1 knockdown on protein phosphorylation and LC3-II levels.A375 cells were transfected with scrambled or TAK1 siRNA (2.5 nmole each). After incubation for 48 hr, the cells were left untreated or treated with A77 1726 for 16 hr. Cells were harvested and analyzed for the indicated proteins by Western blot. **(D)** S6K1 interacts with TAK1. A375 cells seeded in a 6-well plate were incubated in the absence or presence of A77 1726 (200 μM) or rapamycin (20 nM) for 16 hr. Cell lysates were immunoprecipitated with an anti-TAK1 or anti-S6K1 antibody, followed by Western blot analysis with an anti-TAK1 and anti-S6K1 antibodies. Unimmunoprecipitated cell lysates were included as input controls.

S6K1 negatively regulates the activity of TAK1 [40], a serine/threonine kinase that activates JNK [17–20]. TAK1 also phosphorylates and activates LKB1 [21], a tumor suppressor responsible for AMPK T172 autophosphorylation and activation. Here we tested if S6K1 suppression A77 1726 led to the activation of TAK1, subsequently activating AMPK and JNK. As shown in Figure 11A, A77 1726 induced TAK1 phosphorylation in a time- and dose-dependent manner. A77 1726 treatment also led to the presence of multiple bands, probably as a result of ubiquitination (Figure 11A). 5Z-7-oxozeaenol, an inhibitor of TAK1, blocked A77 1726-induced phosphorylation of AMPK, ULK S555, JNK, and Jun phosphorylation, and blocked A77 1726-induced LC3 lipidation (Figure 11B). TAK1 siRNA had similar effect on A77 1726-induced protein phosphorylation and LC3 lipidation (Figure 11C). Co-immunoprecipitation revealed that S6K1 interacted with TAK1, whereas A77 1726 and rapamycin did not increase interaction of these two kinases (Figure 11D). These observations suggest that TAK1 plays a critical role in mediating A77 1726-induced activation of AMPK and JNK.

## DISCUSSION

Leflunomide is a drug with multiple therapeutic potentials, including immunosuppressive, anti-viral and anti-cancer activities [41–43]. Our recent study showed that A77 1726 is an inhibitor of S6K1, and that inhibition of S6K1 activity leads to the feedback activation of the PI-3 kinase pathway [32]. Our present study provides several lines of evidence that A77 1726 was capable of inducing autophagy: 1) A77 1726 increased the levels of LC3-II and the ratio of LC3-II to LC3-I (Figure 1); 2) A77 1726 induced the accumulation of autophagosome puncta (Figure 2); 3) bafilomycin and colchicine increased A77 1726-induced LC3-II expression (Figure 1); 4) AMPK T172 and ULK1 S555 phosphorylation was increased in A77 1726-treated cells (Figure 5). These observations collectively suggest that A77 1726 induces autophagy.

A77 1726 inhibits the activity of at least three types of enzymes: DHO-DHase, protein tyrosine kinases, and S6K1 [26, 27, 32]. Exogenous uridine was unable to block A77 1726-induced LC3-II levels and autophagosome formation, suggesting that A77 1726-induced autophagy is independent of its inhibitory effect on pyrimidine nucleotide synthesis. In support of this notion, brequinar sodium, a much stronger inhibitor of DHO-DHase than leflunomide, was unable to induce LC3-II lipidation (Figure 3D). Activation of AMPK plays a critical role in inducing autophagy [44]. S6K1 knockdown or inhibition of S6K1 activity by PF-4708671 led to AMPK and ULK1 phosphorylation and activation (Figure 7), and subsequent to autophagy (Figure 4). A77 1726 is an inhibitor of S6K1 activity in an *in vitro* kinase assay and in cell culture [32]. AMPK T172 and ULK1 S555 phosphorylation was increased in A77 1726-treated cells (Figure 5). We conclude that A77 1726-induced autophagy is mediated by inhibition of S6K1 activity. During preparation of this manuscript, Chen et al. [45] reported that leflunomide induces autophagy in renal cell carcinoma cell lines. Though A77 1726 inhibits PDGF receptor and Src family tyrosine kinases [26, 27], it has no effect on insulin receptor and IGF-1 receptor but rather stimulates IGF-1/IR-induced PI-3 kinase pathway through S6K1-mediated feedback activation [32]. These observations suggest that A77 1726-induced autophagy is not mediated through IGF-1/insulin receptor or other receptor tyrosine kinases, but rather through inhibition of S6K1 activity. In support of this notion, Blommaart et al. [46] showed that phosphorylation of ribosomal protein S6 is inhibitory for autophagy in isolated rat hepatocytes. Shin et al. [47] reported that silencing S6K1 with siRNA induces autophagy in HEK-293T cells. Consistently, Park et al. [48] reported that PF-4708671 induces autophagy in mouse embryonic fibroblast cells.

**Figure 12:**
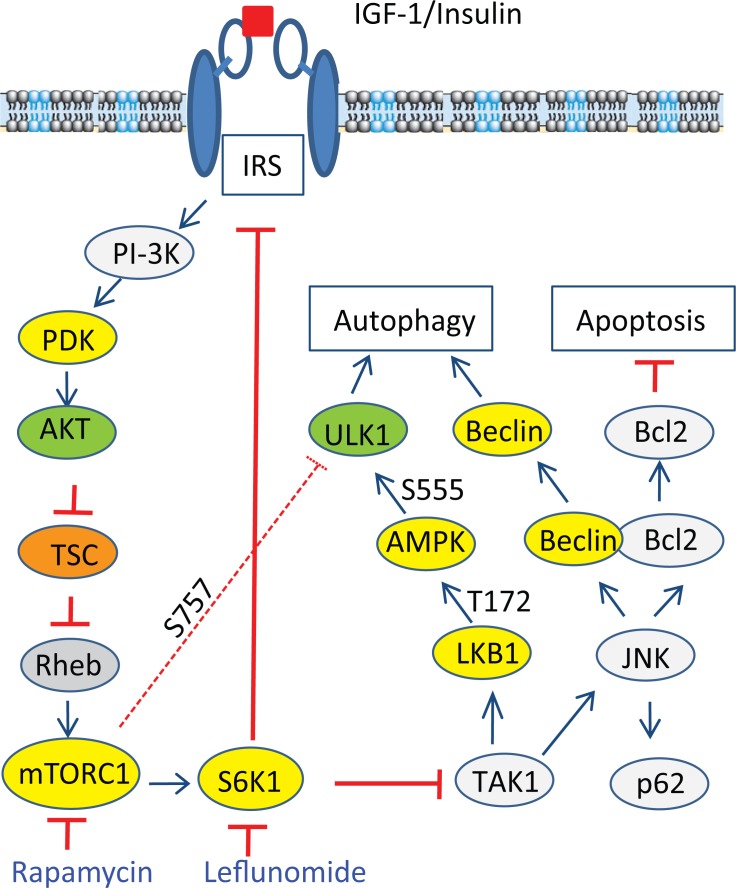
Mechanisms of A77 1726-induced autophagy A77 1726 inhibits S6K1 activity, leading to TAK1 activation that subsequently activating AMPK and JNK. JNK activation leads to increased p62 expression and disrupts Beclin1 and Bcl2 interaction by phosphorylating Bcl2. Although feedback activation of mTOR by A77 1726 leads to ULK1 phosphorylation at S757, it does not dampen ULK1 phosphorylation at S555 and activation by AMPK. AMPK appears to be at the high end of hormone and metabolic signal pathways that modulates nutrient and energy homeostasis through autophagy.

We further analyzed the signaling pathway of S6K1 suppression-induced autophagy. Kim et al. [40] recently showed that in the TLR-signaling pathway, S6K1 negatively regulates the activity of TAK1. Inokuchi-Shimizu et al. [23] reported that TAK1 deficiency leads to the inhibition of starvation-induced AMPK and ULK1 phosphorylation and activation, subsequently suppressing autophagy in the liver of TAK1-deficient mice. These investigators further showed that TAK1 deficiency also compromises rapamycin-induced autophagy in the hepatocytes of TAK1-knockout mice, indicating that TAK1 is partially required for rapamycin-induced autophagy (Figure 12). Consistent with these observations, we found that inhibition of TAK1 activity by 5Z-7-oxozeaenol or by TAK1 siRNA abrogated A77 1726-induced activation of AMPK and JNK, reduced A77 1726-induced LC3 lipidation. These observations collectively suggest that A77 1726 induces autophagy by inhibiting S6K activity, leading to TAK1 activation, which then activates AMPK and JNK (Figure 12). Of note, S6K1 deficiency leads to AMPK activation in the skeletal muscle tissues and myotubes of S6K1-deficient mice due to increased AMP levels and AMP/ATP ratio [9, 11]. It is not clear if A77 1726-induced AMPK activation is also mediated in part by increased AMP levels and AMP/ATP ratios.

ULK1 phosphorylation at S757 by mTOR suppresses its activity and autophagy [6, 7]. In contrast, ULK1 phosphorylation at S555 by AMPK leads to ULK1 activation and autophagy. Several other sites including S317, S777, S638, S467, S556, T575 can also be phosphorylated by AMPK but seem to have only marginal effects on the activity of ULK1 [3, 6]. Since inhibition of S6K1 activity by A77 1726 leads to the feedback activation of the PI-3 and MAP kinase pathways through the IGF-1 receptor [32], feedback activation of mTOR by A77 1726 should suppress autophagy. Surprisingly, though ULK1 was highly phosphorylated at S757 due to mTOR feedback activation, A77 1726 did not inhibit but rather induced autophagy. Moreover, A77 1726 induced AMPK phosphorylation at T172 and ULK1 phosphorylation at S555. These observations suggest that AMPK activation through inhibition of S6K1 activity in A77 1726-treated cells was able to blunt the inhibitory effect of feedback-activated mTOR on ULK1 activity. Our further study showed that rapamycin blocked A77 1726-induced ULK1 S757 phosphorylation but did not further increase autophagy (Figure 3). It appears that, when ULK1 is phosphorylated by AMPK at S555 and possibly other sites [3, 49], ULK1 activity can no longer be restrained by S757 phosphorylation. Consistent with our observations, Shang et al. [50] reported that ULK1 S757 phosphorylation transiently regulates autophagy and does not alter the maximum capacity of autophagy after a prolonged starvation. Kang et al. [7] reported that rapamycin is not very effective at inhibiting ULK1 S757 phosphorylation *in vitro*. Although ULK1 S757 phosphorylation disrupts its interaction with AMPK, we found that mTOR feedback activation and ULK1 S757 phosphorylation did not ablate the interaction between ULK1 and AMPK in A77 1726-treated A375 cells (Figure 5E).

Our present study showed that A77 1726 induced JNK phosphorylation and activation in A375 cells in a time- and dose-dependent manner (Figure 9). SP600125 blocked A77 1726-induced Jun phosphorylation and p62 expression. Consistently, S6K1 knockdown and PF-4708671 also led to increased p62 levels as well as JNK and Jun phosphorylation. These results collectively suggest that induction of p62 expression by A77 1726 is mediated by inhibition of S6K1 activity and subsequent activation of JNK. Puissant et al. [38] reported that resveratrol inhibits the PI-3 kinase pathway and induces autophagy in a K562 chronic myelogenous leukemia cell line. Interestingly, resveratrol also activates JNK and transcriptionally induces p62 expression [38]. JNK phosphorylates Bcl-2 and promotes its dissociation from Beclin, subsequently promoting autophagy [51] (Figure 12).

Our study suggests that increased p62 levels by A77 1726 are due to JNK activation. Interestingly, p62 levels were not decreased in rapamycin-treated A375 cells in which JNK activation was not observed. Although in most cases, induction of autophagy by rapamycin leads to the degradation and decreased levels of p62, Ju et al. [52] reported that rapamycin induces autophagy but does not reduce p62 levels *in vitro* in C2C12 myotubes and *in vivo* in the muscle of rapamycin-treated mice. Kim et al. [53] reported that activation of the MAP kinase pathway can up-regulate p62 transcription. Rapamycin induces the feedback activation of the MAP kinase pathway [54], it is likely that rapamycin increases p62 levels through MAP kinase pathway-induced transcriptional regulation of p62. A77 1726 not only induces the feedback activation of the MAP kinase pathway [32] but also strongly activated JNK (Figure 10), and increased p62 expression much stronger than rapamycin. It appears that JNK activation plays a dominant role in mediating A77 1726-induced p62 expression. However, it remains enigmatic why indirect inhibition of S6K1 activity by rapamycin did not activate JNK.

He et al. [55] reported that AMPK phosphorylates JNK *in vitro*, and that AMPK activation by metformin stimulates its interaction with JNK. However, this supposition is challenged since the amino acid sequences in the activation loop sites of JNK do not contain the AMPK consensus, and AMPK does not have a tyrosine kinase activity [44]. Our study showed that the AMPK inhibitor CC failed to block A77 1726-induced JNK activation, while oligomycin and metformin activated AMPK but had no or little effect on JNK activation (Figure 10), suggesting that AMPK is not responsible for activating JNK. On a cautionary note, since CC is not a very specific inhibitor of AMPK [56], the concentration of the compound C we used was relatively low and appeared not capable of inhibiting the major enzyme involved in autophagy. Furthermore, we found that 5Z-7-oxozeaenol and TAK1 siRNA inhibited A77 1726-induced JNK and Jun phosphorylation, suggesting that A77 1726-induced JNK activation is mediated by TAK1 activation (Figure 12). Consistent with this notion, TAK1 plays a critical role in activating the hematopoietic progenitor kinase-1 (HPK1)-induced JNK activation [17].

In summary, our study showed that inhibition of S6K1 by A77 1726 led to the activation of AMPK and JNK, both of which contributed to A77 1726-induced autophagy. We further showed that AMPK phosphorylation at T172 and activation led to ULK1 phosphorylation at S555, which overcame the inhibitory effect of ULK1 S757 phosphorylation mediated by feedback-activated mTOR. We further showed that A77 1726 activated AMPK and JNK through S6K1 inhibition-mediated TAK1 activation (Figure 12). Our studies reveal a novel mechanism by which the mTOR-S6K1 pathway connects to the AMPK-ULK1 pathway through TAK1 to regulate autophagy (Figure 12).

## MATERIALS AND METHODS

### Reagents

Leflunomide and A77 1726 were kindly provided by Cinkate Corporation (Oak Park, IL). Brequinar sodium (BQR), a potent inhibitor of DHO-DHase, was kindly provided by Dupont Corporate (Wilmington, DE). PLX4720 was purchased from Selleck Chemicals Inc. (Houston, TX). SP600125, U0126, PD98059, and LY294002 were purchased from Cell Signaling Technology (Danvers, MA). Rapamycin was purchased from Cayman Laboratories (Ann Arbor, MI). Bafilomycin, colchicine metformin, 5Z-7-oxozeaenol, PF-4708671, and oligomycin were purchased from Sigma (St. Louis, MO). Anti-actin mAb and PPP were purchased from Santa Cruz Biotechnology, Inc. (Santa Cruz, CA). Antibodies against p62, LC3, ULK1, AMPK, JNK, Jun, AKT, S6K1, S6 and their corresponding phospho-antibodies including ULK1^S555^, ULK1^S757^, AMPK^T172^, AKT^S473^, S6K1^T389^, S6^S235/236^, TAK1^T184/187^ were purchased from Cell Signaling Technology (Danvers, MA). The expression vector encoding RFP-LC3 (pmRFP-LC3) was purchased from OriGene Technologies, Inc. (Rockville, MD).

### Cell lines

A375 is a melanoma cell line with BRAF^V600E^ mutation, wild-type PTEN/PI3KC and p53. MCF-7 is an estrogen receptor-positive breast cancer cell line with PI3KC mutation but with wild-type p53. A375 cells were grown in complete DMEM medium supplemented with 10% fetal bovine serum, streptomycin and penicillin, and L-glutamine. MCF-7 cells were grown in the complete MEM medium supplemented with 10% fetal bovine serum, streptomycin and penicillin, and L-glutamine, non-essential amino acids, and HEPES buffer. C2C12 cells were grown in DMEM supplemented with 10% fetal bovine serum, streptomycin and penicillin, and L-glutamine. For induction of differentiation, the cells were cultured in the complete DMEM medium containing 10% horse serum for 7-10 days. The medium was replenished every three days. All three cell lines were purchased from American Type Culture Collection (Manassas, VA).

### Western blot

Cells grown in 6-well plates were harvested and lysed in NP-40 lysis buffer (50 mM Tris-HCl (pH 8.0), 150 mM NaCl, 1% NP-40, 5 mM EDTA, 10 μg/ml aprotinin, 10 μg/ml leupeptin, and 1 mM phenylmethylsulfonyl fluoride). After incubation on ice for 30 min, the cell lysates were prepared by spinning down at 4°C, 15,000 rpm for 15 min. After electrophoresis and transfer to Immobolin or nitrocellulose membranes, proteins of interest were probed with their specific antibodies, followed by horseradish peroxidase-conjugated goat anti-rabbit IgG and SuperSignal Western Pico enhanced chemiluminescence substrate (Pierce Chemical Co., Rockford, IL).

### S6K1 and TAK1 knockdown

S6K1 siRNA ON-TARGETplus SMARTpool was synthesized by Dharmacon and purchased from Fisher Scientific (Pittsburg, PA). This S6K1 siRNA pool containing three different siRNAs has been previously shown to efficiently suppress S6K1 expression [57, 58]. TAK1 siRNA was purchased from Cell Signaling Technology (Danvers, MA). A scrambled control siRNA was purchased from Life Technologies (Invitrogen Life Technologies, Grand Island, NY). A375 cells seeded in a 6-well plate were transfected with siRNA using Lipofectamine RNAiMAX (Invitrogen Life Technologies, Grand Island, NY) according to the manufacturer’s instruction. After incubation for 48 hr, the cells were harvested and analyzed for S6K1 expression and for the phosphorylation of S6K1, AKT, S6, AMPK, and ULK1 by Western blot.

### RFP-LC3 fluorescence analysis

A375 and MCF-7 cells seeded on coverslips were transiently transfected with RFP-LC3 expression plasmid DNA using FuGENE6 following the manufacturer’s protocol. After incubation for 48 hr, the cells were incubated in the presence of A77 1726 (200 μM), rapamycin (20 nM), leflunomide (200 μM) or PF-4708671 (5 μM). After incubation for 16 hr, the cells were fixed in 100% methanol at -20°C for 10 min. The coverslips were mounted with 50% glycerin in PBS containing 4,6-diamidino-2-phenylindole (0.5 μg/ml; Sigma Chemical Co.). Autophagosomes were examined under a Leica LP8 confocal microscope. The autophagosome puncta were examined under a Nikon fluorescence microscope. To determine the effect of S6K1 knockdown on autophagosome formation, A375 cells were transfected with control or S6K1 siRNA as described above. After incubation for 24 hr, the cells were transfected with RFP-LC3 plasmid DNA again. After incubation for another 48 hr, the coverslips were collected, fixed, and mounted on slides and examined for RFP fluorescence under a fluorescent microscope. Autophagosome puncta in A375 cells treated with various drugs or siRNA transfection were counted in 30 randomly selected fields under a 40X objective in a blinded fashion. Results represent the mean ± SD (standard deviation) from three experiments.

### Immunoprecipitation

A375 cell lysates in NP-40 lysis buffer were incubated at 4°C with the indicated antibody (2 μg/sample) overnight followed by incubation for 2 hr with Protein A/G-conjugated agarose beads that had been blocked with 5% BSA and washed with NP-40 lysis buffter. The agarose beads were washed 3 times with NP-40 lysis buffer. Immunoprecipitates were analyzed by Western blot with specific antibodies.

### Statistical analysis

The differences in the number of puncta in A375 cells treated with various drugs were statistically analyzed by using an unpaired Student *t* test. A *p* value of <0.05 was considered statistically significant. All statistics was performed with SigmaPlot 11 software (Systat Software, Inc, San Jose, CA).

## Abbreviations

AMPK: AMP-activated protein kinase
BQR: brequinar sodium
CC: compound C
DHO-DHase: dihydroorotate dehydrogenase
JNK: c-Jun N-terminal kinase
S6K1: p70 S6 kinase
RFP: red fluorescence protein
